# Inflammatory bowel disease patients believe cannabis and cannabidiol oil relieve symptoms

**DOI:** 10.20935/acadmed7773

**Published:** 2025-06-17

**Authors:** Ayati Lala, Alexander Rodriguez-Palacios, Fabio Cominelli, Abigail Raffner Basson

**Affiliations:** 1 Department of Neurosciences, Case Western Reserve University, Cleveland, OH 44106, USA.; 2 Division of Gastroenterology and Liver Diseases, Case Western Reserve University School of Medicine, Cleveland, OH 44106, USA.; 3 Digestive Health Research Institute, University Hospitals Cleveland Medical Center, Cleveland, OH 44106, USA.; 4 Mouse Models, Silvio O’Conte Cleveland Digestive Diseases Research Core Center, Cleveland, OH 44106, USA.; 5 Department of Nutrition, Case Western Reserve University School of Medicine, Cleveland, OH 44106, USA.

**Keywords:** cannabis, cannabidiol oil, Crohn’s disease, ulcerative colitis, inflammatory bowel disease

## Abstract

**Background:**

Patients with Inflammatory bowel disease (IBD) often seek alternative therapies for symptom management. This study investigates the perceptions, consumption patterns, and reported outcomes of cannabis and cannabidiol (CBD) oil use among IBD patients and controls.

**Methods:**

A 37-question survey was administered to 139 participants (IBD patients, *n* = 93; control/non-IBD participants, *n* = 33) to assess usage frequency and beliefs regarding cannabis and CBD oil as treatment for IBD. The survey also evaluated the impact of these substances on IBD symptoms, quality of life, and opioid use.

**Results:**

Cannabis consumption was higher in IBD patients (57, 53.8%) than controls (15, 45.5%) with both groups strongly supporting medical cannabis use (IBD; 92, 86.8% vs. controls; 29, 84.9%). Most IBD patients believed cannabis (67, 63.2%), CBD oil (60, 56.6%), corticosteroids (77, 73.3%), and biologics/immunosuppressants (85, 81.0%) had a somewhat-extremely beneficial effect in relieving IBD symptoms. Over 50% of IBD cannabis users reported relief from abdominal pain, other pain, stress, anxiety, depression, and nausea/vomiting, with Crohn’s disease patients experiencing significantly more relief than ulcerative colitis patients for certain symptoms (*p* < 0.05). Notably, 19.4% of IBD patients reported decreased opioid use, and 14.5% reported induced remission with cannabis or CBD oil.

**Conclusions:**

Consumption of cannabis and CBD oil was perceived as beneficial for relieving IBD, with many reporting significant symptom relief from using these substances. The strong support of cannabis and CBD oil as medical treatments and therapeutic effects highlights the potential for cannabis and CBD oil as treatments in IBD.

## 1. Introduction

Inflammatory bowel disease (IBD), including Crohn’s disease (CD) and ulcerative colitis (UC), is a chronic disease of episodic inflammation of the gastrointestinal tract, in which current therapeutic strategies often do not lead to a complete resolution of symptoms [1, 2]. Thus, there has been increasing interest in alternative treatment options, one of which is Cannabis sativa, commonly known as marijuana [3, 4]. The cannabis plant contains cannabinoids, such as delta-9-tetrahydrocannabinol (THC) and cannabidiol (CBD), which interact with the human endocannabinoid system (ECS) [5, 6].

The ECS is a complex cell-signaling system involved in regulating homeostasis and modulating several physiological functions such as pain, appetite, mood, and stress response [5]. It comprises endogenous cannabinoids, which activate our cannabinoid receptors (CB1 and CB2) and enzymes responsible for cannabinoid synthesis/degradation [3, 5, 6]. Cannabis-derived cannabinoids can influence the ECS by mimicking endogenous cannabinoids and activating the same receptors [3, 5, 6].

Recent preclinical studies have suggested that cannabinoids, including CBD and THC, may reduce gastrointestinal inflammation and modulate intestinal motility [4, 7]. The presence of cannabinoid receptors in the gut, along with the anti-inflammatory effects of some cannabinoids, has led researchers to explore the potential for therapeutic applications of cannabis in the treatment of IBD [5, 7–10].

Animal studies of the chemistry and physiology of cannabinoids have shown potential anti-inflammatory, antidiarrheal, and nociceptive-limiting effects, paralleling the growing interest in cannabis as a treatment option for IBD [5, 11]. Human studies have also indicated that there may be a benefit in controlling IBD symptoms and improving quality of life [5]. Additionally, anecdotal reports and observational studies have highlighted potential benefits in reducing disease activity and managing symptoms like abdominal pain, cramping, joint pain, and diarrhea [8–10, 12–14]. In a 2014 survey of 292 IBD patients, 16.4% of current and past marijuana users reported using marijuana for disease symptoms, with a majority claiming it was “very helpful” for relief [9]. Several small-scale clinical trials have also shown that cannabis or cannabinoids may improve quality of life, reduce disease activity in CD patients, and reduce inflammation in UC patients, though results are not always significant [4, 14]. Long-term effects remain unclear, with some studies suggesting cannabis could improve long-term prognosis while others suggest chronic use may increase surgery risk in CD [1, 8, 15, 16].

Complex interactions among cannabis, the ECS, and the pathophysiology of IBD underlie that the use of cannabis and CBD oil by IBD patients needs to be further investigated, given that few clinical trials are evaluating its effectiveness in the management of IBD. This study aimed to survey the consumption patterns and beliefs of IBD and non-IBD controls in our university hospital setting regarding their use of cannabis and CBD oil to treat IBD symptoms as alternative therapies. While many IBD patients perceive benefits from cannabis use, there is a significant gap between patient experiences and clinical evidence, highlighting the need for more comprehensive research in this area. This study contributes to the growing body of knowledge on alternative IBD treatments and provides valuable insights into patient perspectives, which can inform future clinical trials and treatment strategies.

## 2. Materials and methods

### 2.1. Study design and participants

This study was designed as a survey-based analysis to assess perceptions of cannabis use among individuals with inflammatory bowel disease (IBD) and a control group. This survey-based cross-sectional study included 139 participants: 106 IBD patients and 33 non-IBD controls, ages 18–69 years. Of the 139 total participants, 66 IBD and 21 non-IBD controls reported cannabis or CBD. Controls were identified from the same population as the IBD patients (friends and family members). Among the participants, 132 were from Ohio and 7 were from other states. All participants were above 18 years old and able to provide informed consent.

### 2.2. Survey

The study employed an anonymous, self-administered online questionnaire to evaluate participants’ knowledge, perceptions, and beliefs regarding the use and effects of cannabis and CBD oil as alternative therapies for digestive health. The questionnaire contained 25 questions, with 12 additional questions only to be answered by participants who responded ‘yes’ to having used cannabis or CBD oil in the past (37 total questions).

Demographic information including age, sex, ethnic background, education level, employment status, and income levels was obtained for all participants. For IBD participants, information on disease diagnosis (CD, UC, indeterminate), disease duration, and symptoms in the last two weeks and six months was obtained.

Participants who responded yes to having used cannabis or CBD oil were then asked questions about frequency, mode and patterns of use, their subjective benefits on IBD symptoms, and their perceived side effects. As the questionnaire was created before cannabis legalization in Ohio, questions were asked about access to cannabis/CBD, whether healthcare providers had recommended its use, and personal motives for cannabis use.

The questionnaire included multiple choice, open ended, and ranking questions based on a scale from 1 to 5. Ranking scales were defined as (i) 1 = ‘not beneficial’ to 5 = ‘extremely beneficial’, (ii) 1 = ‘not important’ to 5 = ‘very important’, (iii) 1 = ‘strongly support’ to 5 = ‘do not support’, or (iiii) 1 = ‘no relief’ to 5 = ‘complete relief’ (Table S1). All questions had a ‘I do not know’ or ‘I do not use’ option.

Informed consent was obtained electronically and was required for participants to gain access to the survey. The survey recruitment email was sent out a maximum of 3 times. The study’s survey, recruitment protocols, and objectives were approved by the Ethics Committee of University Hospitals Cleveland Medical Center (STUDY20230400, approved on 2023 July 3).

### 2.3. Statistics

To characterize the study population, we conducted a descriptive analysis of participants’ demographics, IBD-specific information, and patterns of cannabis and CBD oil consumption. Univariate analysis was conducted to evaluate associations between variables and participant groups (IBD patients and controls). Data are expressed as frequencies for categorical variables. To assess differences in profiles, perceptions, and usage patterns between IBD patients and controls, as well as between CD and UC patients, the unadjusted relationships between these variables were analyzed using Fisher’s exact or Chi-square tests for count data across categorical variables. For questions involving Likert scales (e.g., perceived benefits of treatments, symptom relief), responses were treated as ordinal data. For all statistical tests, a *p*-value of 0.05 or less was considered an indicator of a significant difference. Statistical analysis was carried out using GraphPad Prism 10.3.1.

## 3. Results

### 3.1. Demographics and reported use of cannabis and cannabidiol oil

The questionnaire was answered by a total of 139 individuals, of whom 106 (76.3%) had IBD and 33 (23.7%) were non-IBD controls. Of the IBD participants, 66 (47.5%) had Crohn’s disease (CD), 26 (18.7%) had ulcerative colitis (UC), 9 (6.5%) reported having both CD and UC, and 5 (3.6%) had indeterminate IBD. For the purpose of this study and analysis, CD, UC, and indeterminate IBD were grouped as IBD. Additional reported comorbidities included the following: irritable bowel syndrome (IBS); IBS [*n* = 19; 11 (7.9%) with IBD and 8 (5.8%) controls], diabetes [*n* = 14; 7 (5.0%) with IBD and 7 (5.0%) controls], and cancer [*n* = 2; 1 (0.72%) with IBD and 1 (0.72%) controls].

Table 1 outlines the demographic and socioeconomic characteristics of the study population. There were no significant differences in the demographic characteristics between IBD patients and controls (*p* > 0.05), with the majority of IBD patients (*n* = 67/100, 67%) being diagnosed more than 11 years ago, followed by diagnoses between 1 and 5 years (vs. *n* = 17/100, 17%) and 6–10 years ago (*n* = 16/100, 16%).

### 3.2. Knowledge, access, and personal usage of cannabis and cannabidiol product

Table 2 summarizes the participants’ knowledge, access, and personal use of cannabis and CBD oil. Overall, the majority of study participants knew what cannabis is (IBD; 105/106, 99.1% vs. controls; 32/33, 97.0%) and CBD oil (IBD; 101/106, 95.3% vs. controls; 33/33,100%) (Fisher’s exact *p* = 0.420, *p* = 0.339, respectively).

In terms of being able to access cannabis or CBD products, there was no difference between IBD patients and controls for both cannabis (IBD; 45/106, 42.5% vs. controls; 13/33, 39.4%) and CBD oil (IBD; 57/106, 53.8% vs. controls; 19/33, 57.6%). Of note, 20 (18.9%) IBD patients and 3 (9.1%) controls reported having cannabis recommended to them by a medical professional. Approximately half of both the IBD and control groups reported cannabis use (IBD; 57, 53.8% vs. controls; 15, 45.5%); however, significantly fewer IBD patients reported using CBD oil compared to controls (IBD; 43, 40.6% vs. controls; 21, 63.6%, Chi-square *p* = 0.020) (Table 2).

Table 3 provides a detailed overview of the frequency and modes of cannabis or CBD oil use among IBD patients and controls. This question did not differentiate between cannabis and CBD oil use. Both IBD and control participants reported mainly using cannabis or CBD oil between the ages of 18 and 30 years (IBD: 28/62, 45.2% vs. controls: 9/21, 42.9%, *p* = 0.572), compared to before the age of 18 years (IBD: 16/62, 25.8% vs. controls: 3/21, 14.3%, *p* = 0.374), or after the age of 30 years (IBD: 16/62, 25.8% vs. controls: 9/21, 42.9%, *p* = 0.141). When asked how many times IBD patients and controls had used/consumed cannabis or CBD oil in the past 30 days, their answers ranged from none to multiple times every day. Among the 62 IBD participants and 21 controls who reported using these substances, most reported using less than once a day (IBD: 22/62, 35.5% vs. controls: 10/21, 47.6%, *p* = 0.323), followed by once or twice a day (IBD: 10/62, 16.1% vs. controls: 4/21, 19.1%, *p* = 0.686), and three or more times a day (IBD: 6/62, 9.7% vs. controls: 2/21, 9.5%, *p* = 1.000). Twenty-three (37.1%) IBD participants and four (19.0%) controls reported not currently using these substances (*p* = 0.179). Modes of use reported among both groups included edibles, smoking, oils, sublingual sprays, and tinctures.

### 3.3. Inflammatory bowel disease patients believe cannabis and cannabidiol oil relieves gastrointestinal symptoms

**Figure 1 •:**
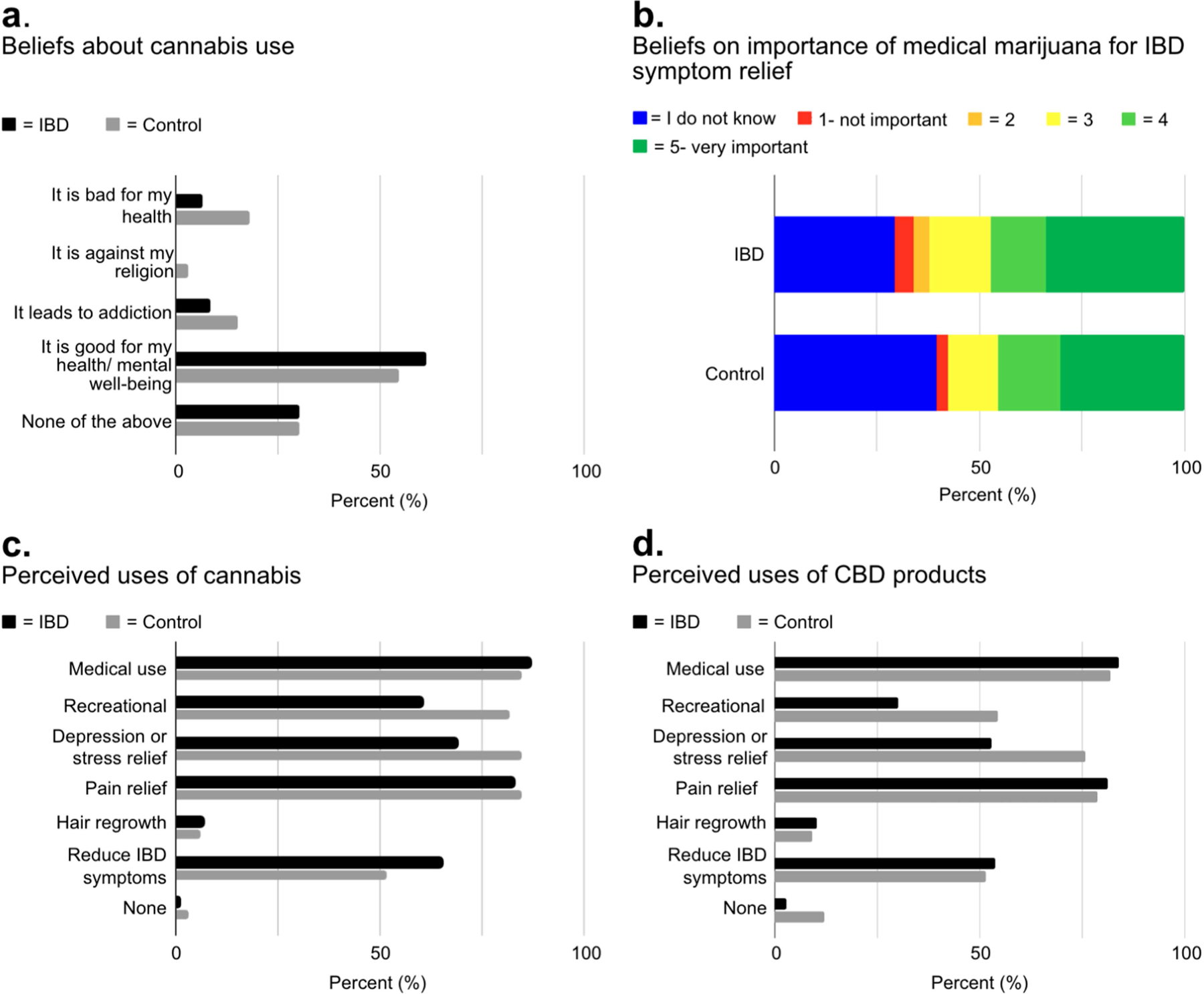
Perceptions and usage of cannabis and CBD products. (**a**) Perceptions of cannabis use on health and well-being. (**b**) Perceived importance of medical marijuana for IBD symptom relief. (**c**,**d**) Perceptions of potential applications for cannabis and CBD oil. IBD = Inflammatory bowel disease; CBD = Cannabidiol.

Figure 1 illustrates the perceived effects of cannabis on health among IBD patients and control participants.

The majority of IBD (*n* = 65/106, 61.3%) and control participants (*n* = 18/33, 54.6%) believed cannabis use was good for their health/mental well-being. By comparison, only a small percentage of IBD patients (7, 6.6%) and controls (6, 18.2%) believed it was bad for their health or led to addiction (IBD; 9, 8.5%, controls; 5, 15.2%) (Figure 1a). IBD and control participants then ranked their beliefs on the importance of medical marijuana for symptom relief in IBD patients, on a scale of 1 to 5, with 1 = ‘not important’ and 5 = ‘very important’. A majority in both groups ranked medical marijuana between a 3 and 5 regarding its importance for symptom relief (IBD; 66, 62.3% vs. controls; 19, 57.6%) (Figure 1b). Both IBD and control participants largely supported the use of cannabis and CBD oil for medical use/purposes (IBD; 92, 86.8% vs. controls; 28, 84.9%). Cannabis was also viewed favorably for depression/stress relief (IBD; 73, 68.9% vs. controls; 28, 84.9%) and pain relief (IBD; 88, 83.0% vs. controls; 28, 84.9%). Likewise, CBD oil was widely considered beneficial for medical use (IBD; 89, 84.0% vs. controls; 27, 81.8%), depression/stress relief (IBD; 56, 52.8% vs. controls; 25, 75.8%), and pain relief (IBD; 86, 81.1% vs. controls; 26, 78.8%). Overall, a majority in IBD and controls groups believed cannabis (IBD; 69, 65.1% vs. controls; 17, 51.5%) and CBD oil (IBD; 57, 53.8% vs. controls; 17, 51.5%) could be used to reduce IBD symptoms (Figure 1c,d).

**Figure 2 •:**
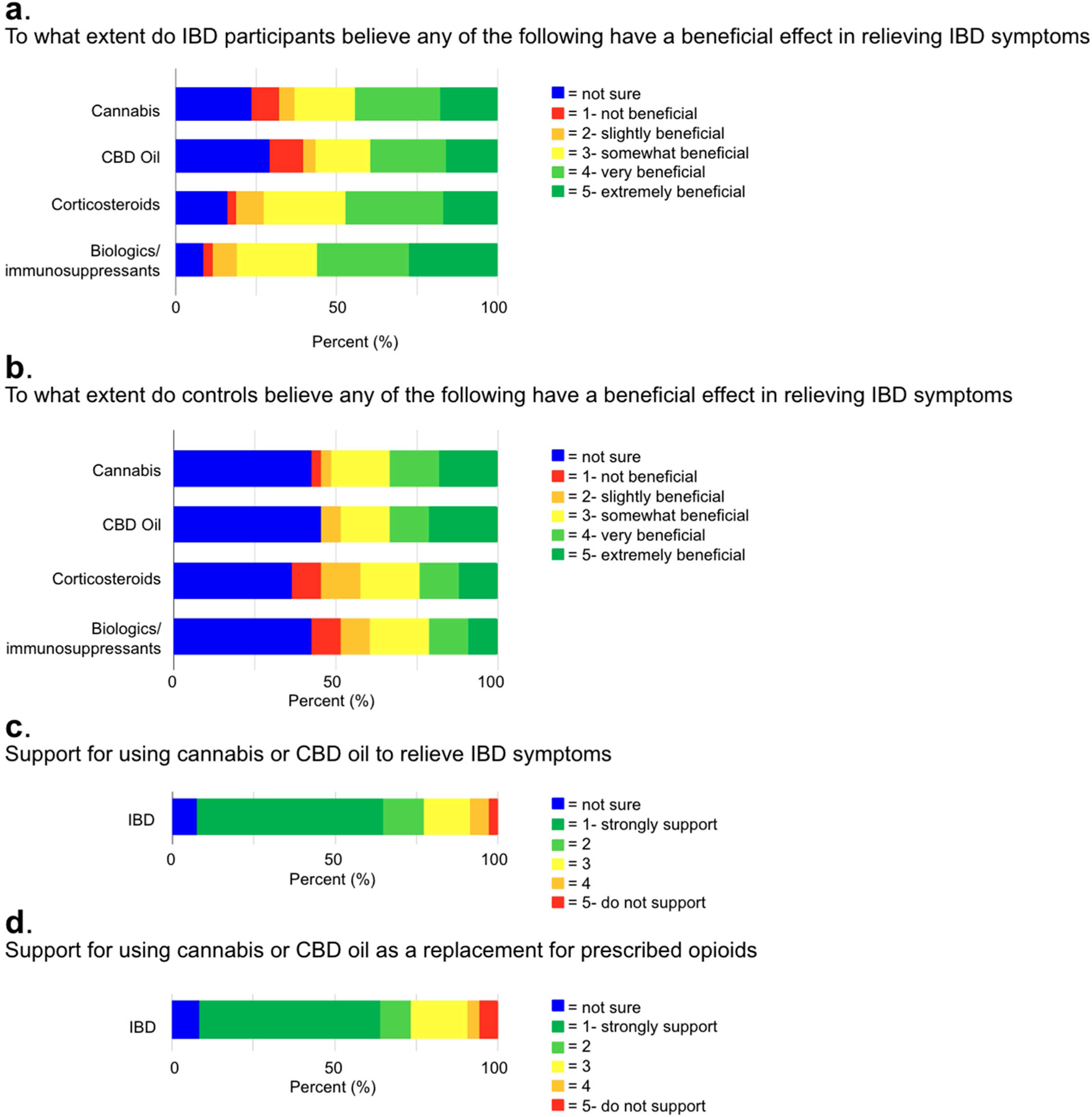
Perceptions and support for cannabinoid-based and conventional therapies in IBD management. (**a**,**b**) Perceived benefits of cannabis, CBD oil, corticosteroids, and biologics/immunosuppressants in relieving IBD symptoms. (**c**,**d**) IBD patients’ support for cannabis and CBD oil use in IBD treatment and as opioid alternatives. IBD = Inflammatory bowel disease; CBD = Cannabidiol.

Figure 2 shows participants’ perceptions of cannabis and other therapies for managing IBD symptoms: cannabis, CBD oil, corticosteroids, and biologics/immunosuppressants. Participants rated the perceived benefit of each therapy on a scale from 0 to 5: ‘not sure’ = 0, ‘not beneficial’ = 1, ‘slightly beneficial’ = 2, ‘somewhat beneficial’ = 3, ‘very beneficial’ = 4, and ‘extremely beneficial’ = 5. This assessment was conducted for both IBD and control groups, given the generally positive perception and support for cannabis and CBD oil use in IBD symptom management observed among participants. Many IBD and control participants believed cannabis (IBD; 67, 63.2% vs. controls; 17, 51.5%), CBD oil (IBD; 60, 56.6% vs. controls; 16, 48.5%), corticosteroids (IBD; 77/105, 73.3% vs. controls; 14, 42.4%), and biologics/immunosuppressants (IBD; 85/105, 81.0% vs. controls; 13, 39.4%) had a somewhat beneficial to extremely beneficial (ranked 3–5) effect in relieving symptoms of IBD (Figure 2a,b). Participants were then asked about their support for using cannabis and CBD oil to manage IBD symptoms. Participants ranked their support on a scale from 0 to 5, where 0 meant ‘not sure,’ 1 meant ‘strongly support,’ and 5 meant ‘do not support.’ The majority of both IBD (73/105, 69.5%) and control participants (20/33, 60.6%) ranked their support for using cannabis and CBD oil to manage IBD symptoms as a 1 or 2 (strongly support) (Figure 2c). When asked how strongly they would consider or support using cannabis or CBD oil as a replacement for prescribed opioids, the majority of both IBD (68/105, 64.8%) and controls (27/33, 81.8%) rated their support as a 1 or 2 (strongly support) (Figure 2d).

### 3.4. Inflammatory bowel disease patients report symptom relief with cannabis and cannabidiol product use

**Figure 3 •:**
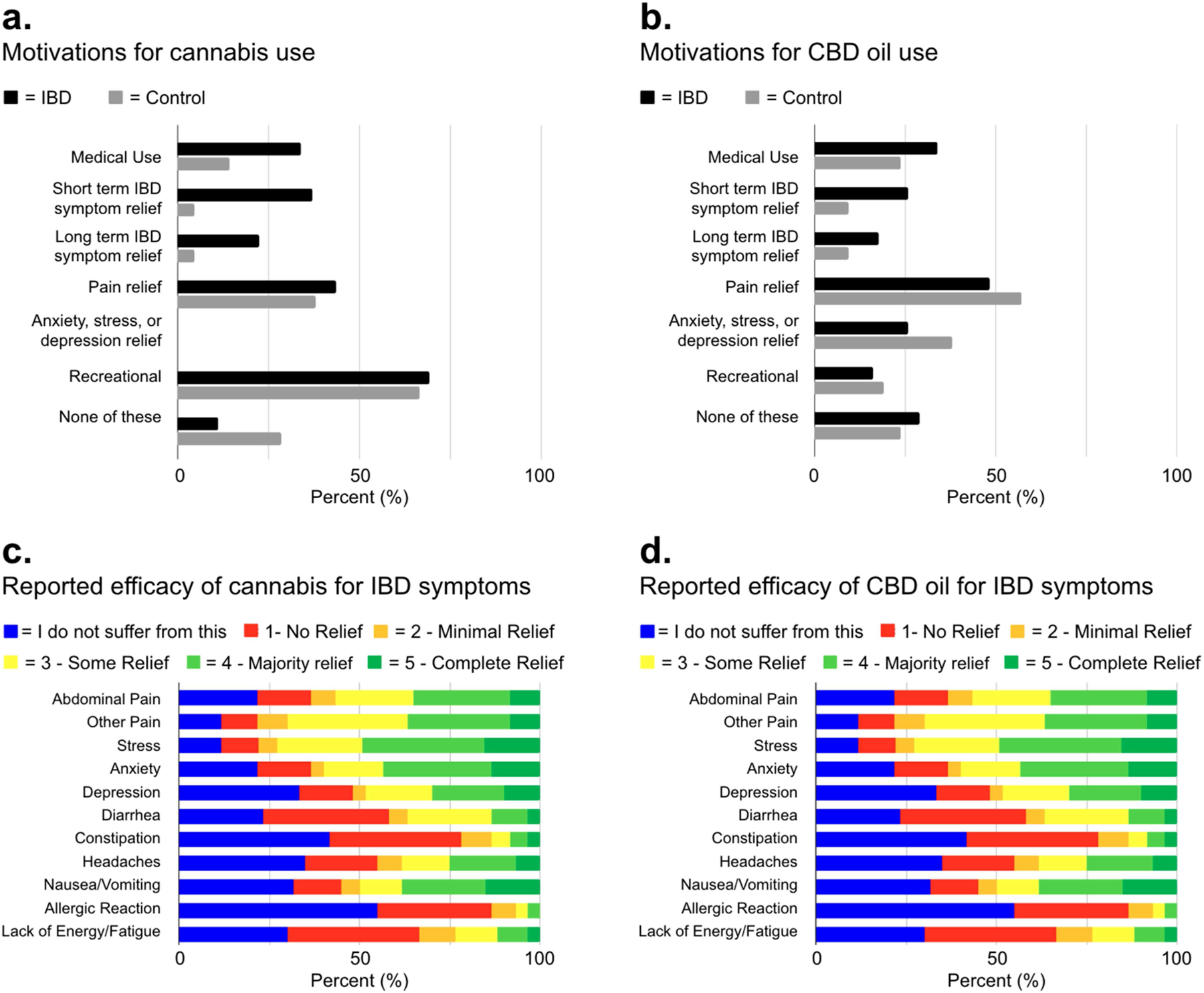
Motivations and efficacy of cannabis and CBD oil use in IBD management. (**a**,**b**) IBD and control group’s reasons for cannabis and CBD oil use. (**c**,**d**) IBD patients’ relief efficacy of cannabis and CBD oil for various IBD symptoms. IBD = Inflammatory bowel disease; CBD = Cannabidiol.

The majority of IBD and control participants reported currently using cannabis/CBD products [IBD; 66/106 (62.3%), controls; 21/33 (63%)]. For the IBD patients using cannabis (*n* = 66), the primary reasons for use were recreational (43, 69.4%), followed by pain relief (27, 43.6%), short-term IBD symptom relief (23, 37.1%), medical use (21, 33.9%), and long-term IBD symptom relief (14, 22.6%). Similarly, reasons for cannabis use among controls were mainly recreational (14, 66.7%), followed by pain relief (8, 38.1%) and medical use (3, 14.3%) (Figure 3a).

For the IBD patients using CBD oil (*n* = 66), the primary reasons for use were for pain relief (30, 48.4%), medical use (21, 33.9%), short-term IBD symptom relief (16, 25.8%), anxiety/stress/depression relief (16, 25.8%), and long-term IBD symptom relief (11, 17.7%). Similarly, controls (*n* = 21) reported CBD oil use was primarily for pain relief (12, 57.1%), anxiety/stress/depression relief (8, 38.1%), medical use (5, 23.8%), recreational use (4, 19.1%), short-term IBD symptom relief (2, 9.52%), and long-term IBD symptom relief (2, 9.52%) (Figure 3b).

Participants were asked to rank the effectiveness of cannabis and CBD oil in relieving symptoms such as abdominal pain, other pain, stress, anxiety, depression, diarrhea, constipation, headaches, nausea/vomiting, allergic reactions, and lack of energy/fatigue. The ranking scale ranged from 0 to 5, where 5 = ‘complete relief,’ 4 = ‘majority relief,’ 3 = ‘some relief,’ 2 = ‘minimal relief,’ 1 = ‘no relief,’ and 0 = ‘I do not suffer from this.’

Among the IBD participants who used cannabis (60 responses), more than half reported experiencing ‘some’, ‘majority’, or ‘complete’ relief (ranking 3–5) for abdominal pain (34, 56.7%), other pain (42, 70.0%), stress (43, 72.9%), anxiety (36, 60.0%), and nausea/vomiting (30, 50.0%) followed by depression (29, 48.3%), headaches (23, 38.3%), diarrhea (22, 36.7%), increased energy (14, 23.3%), constipation (8, 13.3%), and allergic reactions (4, 6.7%). Note, despite the fact that none of the IBD patients reporting using cannabis specifically for stress or anxiety relief, 43 (72.9%) reported stress relief and 36 (60.0%) reported a reduction in anxiety (Figure 3c). CBD oil use (58 responses) appeared to have a lesser effect on IBD symptoms in IBD patients with ratings of ‘some’, ‘majority’, or ‘complete’ relief (scores of 3–5) in the following areas: for other pain (34, 56.7%), stress (23, 39.7%), abdominal pain (21, 36.2%), anxiety (19, 32.8%), depression (18, 31.0%), nausea/vomiting (14, 24.1%), headaches (13, 22.4%), diarrhea (12, 20.7%), increased energy (11, 19.0%), constipation (3, 5.2%), and allergic reactions (2, 3.4%) (Figure 3d).

Among the control participants who used cannabis (21 responses), they reported experiencing ‘some’, ‘majority’, or ‘complete’ relief (scores of 3–5) for stress (12, 57.1%), anxiety (10, 47.6%), other pain (9, 42.9%), depression (7, 33.3%), abdominal pain (4, 19%), nausea/vomiting (4, 19%), headaches (4, 19%), lack of energy/fatigue (3, 14.3%), diarrhea (1, 4.8%), constipation (1, 4.8%), and allergic reactions (0, 0%). Similarly, CBD oil use (21 responses) appeared to have a lesser effect in the control group with ratings of ‘some’, ‘majority’, or ‘complete’ relief (scores of 3–5) in the following areas: for other pain (12, 57.1%), stress (10, 47.6%), anxiety (8, 38.1%), depression (6, 28.6%), abdominal pain (4, 19%), headaches (4,19%), nausea/vomiting (3, 14.3%), headaches (13, 22.4%), lack of energy/fatigue (3, 14.3%), constipation (2, 9.5%), diarrhea (1, 4.8%), and allergic reactions (1, 4.8%).

We then compared cannabis and CBD oil use between CD (*n* = 41 for cannabis, *n* = 39 for CBD oil) and UC (*n* = 11) participants. For cannabis use, significantly more CD participants reported relief of abdominal pain, anxiety, and depression compared to UC individuals (*p* = 0.007, *p* = 0.004, and *p* = 0.007, respectively). No significant differences were observed between CD and UC for other pain, stress, diarrhea, constipation, headaches, nausea/vomiting, allergic reactions, and lack of energy/fatigue. For CBD oil use, significantly more CD participants reported relief from abdominal pain (*p* = 0.010). No significant differences were found between CD and UC participants for any other symptoms (Table 4).

Table 5 shows an overview of the reported effects of cannabis or cannabidiol (CBD) oil use on weight and appetite among IBD patients and controls. Among the participants, the most reported side effect was increased appetite (IBD: 39/62, 62.9% vs. controls: 8/21, 38.1%, *p* = 0.047), followed by weight gain (IBD: 13/62, 21% vs. controls: 1/21, 4.8%, *p* = 0.104). Weight loss was infrequently reported (IBD: 1/62, 1.6% vs. controls: 1/21, 4.8%, *p* = 0.444), and notably, no participants in either group reported decreased appetite (IBD: 0/62, 0% vs. controls: 0/21, 0%, *p* = 1.000).

Additionally, a significant proportion of IBD patients reported decreased opioid usage or the induction of remission as a result of cannabis or CBD oil use when compared to those who reported having no effect (21, 33.9% vs. 46, 74.2%, respectively, Chi-square *p* < 0.000001). Very few IBD patients experienced increased opioid dosage or increased intestinal side effects (2, 3.2% vs. 46, 74.2%, respectively, Fisher’s exact *p* < 0.000001) (Table 6). This highlights that a significant subset of IBD patients who use cannabis or CBD products experience positive outcomes with regard to their disease.

## 4. Discussion

This survey-based study provides insight into the knowledge, perceptions, and beliefs of IBD patients seen in our hospital setting regarding the use of cannabis and CBD oil as alternative therapies. Notably, both IBD patients and controls showed strong support for medical uses of cannabis and CBD oil. The beliefs on the efficacy of cannabis and CBD oil are comparable to that of prescribed medications (e.g., corticosteroids and biologics/immunosuppressants), suggesting that cannabis may be perceived as equally effective. These results show that there exists a strong belief that these substances could be favorable to various IBD symptom relief, including abdominal pain, diarrhea, anxiety, and inflammation.

Of note, a substantial portion of the IBD patients enrolled in this study reported personal experiences using cannabis (54%) and CBD oil (41%) in various contexts of use, including medical use, symptom relief, pain management, and mental health support. A large proportion, 63%, of IBD participants reported that cannabis had a somewhat, very, or extremely beneficial effect in relieving their IBD symptoms, while 57% held this belief about CBD oil. These results are consistent with several studies, including Lal et al. (2011), who found that 33–50% of lifetime cannabis users with IBD specifically used it for symptom relief, reporting improvements in abdominal pain, diarrhea, and appetite [10, 17]. Similarly, Ravikoff Allegretti et al. (2013) noted that 32% of lifetime cannabis users among IBD patients in the U.S. used it for symptom management, particularly for pain and appetite, with many expressing interest in cannabis-based treatments [9, 10, 17]. These self-described benefits may also contribute to the generally favorable perception of cannabis and CBD oil within the IBD community.

Interestingly, we found that IBD patients were more likely to have used cannabis or CBD oil for short-term symptom relief (37% and 26%, respectively) compared to long-term symptom relief (23% and 18%, respectively). Such a pattern indicates that, among IBD patients, these substances are considered to be more effective in the management of acute symptoms rather than in the long-term management of the disease. However, the survey did not assess dosages or concentrations used, limiting our ability to explore dose–response relationships. Given that cannabinoid effects can vary with dosage and frequency, future research should include quantitative tracking and possibly cannabinoid blood level analysis to enhance the objectivity of findings. Our study also did not distinguish consistently between cannabis and CBD oil in all questions, potentially masking substance-specific effects. Future surveys should be designed to differentiate between these products to allow for more precise analysis of perceived benefits and usage patterns.

Mechanistically, cannabinoids are believed to reduce inflammation in IBD via modulation of the endocannabinoid system, particularly CB1 and CB2 receptors found in the gastrointestinal tract. Activation of these receptors may influence cytokine release, gut permeability, and motility. Recent studies have further shown that cannabidiol (CBD) can modulate intestinal barrier function, alter the gut microbiome, and impact cytokine expression, mechanisms that may underlie its potential therapeutic effects in gastrointestinal disorders, including IBD [18]. Additionally, different modes of administration, such as oral ingestion versus topical or rectal formulations, can result in variable absorption and efficacy. Our survey did not collect detailed information on administration route, which should be addressed in future work.

Notably, our study indicated that while IBD patients perceived such substances as helpful, they used them in addition to conventional treatments, which could be indicative that some patients consider cannabis and CBD oil as complementary, not alternative therapies. Nevertheless, more research is needed regarding the long-term implications of cannabis and CBD oil use in IBD. A recent 2024 longitudinal study involving cannabis-based medicinal products reported sustained improvements in health-related quality of life over 18 months among IBD patients, highlighting the potential of cannabinoids in chronic symptom management [19]. However, a 2025 meta-analysis found that while patients report improved quality of life, objective clinical markers such as endoscopic activity and inflammatory levels did not significantly improve, underscoring the need for further trials that integrate subjective outcomes with clinical endpoints [20]. Interestingly, a few participants reported recreational use of CBD oil, which may reflect a misunderstanding of its non-psychoactive properties, which suggests potential gaps in their self-reported knowledge.

It is noteworthy to consider that 20% of IBD patients who used cannabis or CBD oil reported weight gain and increased appetite. Although these effects may be beneficial in some patients struggling with weight loss or lack of appetite, they may also raise concerns regarding metabolic consequences within the context of an increased risk of obesity and metabolic disorders in IBD. Interestingly, 33.9% of IBD patients reported a decrease in opioid dosage when using cannabis or CBD oil suggesting their possible role in mitigating opioid dependence in these patients. However, a few patients also showed increased intestinal side effects or required higher doses of opioids with the use of cannabis/CBD oil. Stress and allergy relief were also self-reported and not corroborated by biochemical validation. In addition, a 2025 study on women with IBD found that CBD use significantly improved nausea, pain, and appetite, suggesting potential sex-specific therapeutic responses [21]. Future studies should stratify outcomes by gender to better elucidate differential responses to cannabinoid-based interventions.

This study has several limitations that should be considered when interpreting the findings. As a cross-sectional study relying on self-reported data and a non-validated questionnaire, the results are exploratory and hypothesis-generating rather than causal. The overlap between medical and recreational motivations also made it difficult to isolate cannabis use specifically for therapeutic purposes. The control group was primarily recruited from friends and family members of IBD patients, which may have introduced selection bias. These individuals are more likely to possess prior knowledge of IBD or be exposed to related discussions around cannabis use. Such familiarity could have influenced their responses, particularly on questions concerning access, beliefs, or comprehension terminology. Future studies should consider recruiting control participants from the general population to reduce this potential bias. Additionally, the control group was smaller than the IBD group, and the majority of participants were from Ohio, limiting statistical power and the generalizability of subgroup comparisons. The overall modest sample size, particularly the small number of participants with UC, further limited subgroup analyses. Another limitation is the lack of data on dosage and frequency of cannabinoid use, which precluded the analysis of dose–response relationships. All findings were based on self-reported perceptions without biochemical or clinical validation. Incorporating objective measures such as biomarker analysis or clinician evaluations would enhance clinical relevance of future research. Despite these limitations, the high level of endorsement of cannabis and CBD oil as a medical treatment (IBD: 86.79%, controls: 84.85%) combined with internal consistency across responses and the use of control questions, suggests that the observed perceptions are unlikely to be attributable solely to sampling bias.

In summary, this study has shown increasing interest and positive perceptions by IBD patients toward the use of cannabis and CBD oil as complementary or alternative therapies for symptom management. While our findings suggest a patient-reported association between cannabinoid use and symptom relief, they should be interpreted as exploratory and hypothesis-generating. Future randomized, controlled studies are needed to determine efficacy, safety, and long-term outcomes. Additionally, efforts should be made to address potential concerns and misconceptions surrounding the use of these substances, particularly in the context of adverse effects and long-term implications for disease management.

## 5. Conclusions

This study highlights the common perception among IBD patients that cannabis and CBD oil are effective therapeutic agents for symptom management, in spite of the lack of conclusive clinical evidence. The findings indicate that a significant proportion of IBD patients use cannabis, notice symptom relief, and prefer its therapeutic use. However, variations between patient accounts and clinical trials provide justification for large-scale, controlled trials to validate the efficacy, safety, and long-term outcome of cannabis-based therapy in IBD treatment. As interest in alternative treatments gains momentum, these results can influence future clinical trials, guide healthcare professionals in patient counseling, and be included in altering treatment modalities. By filling this gap between the medical evidence and patient-reported benefit, this study highlights the need for continued investigation into the therapeutic potential of cannabis and CBD oil as IBD therapies.

## Supplementary Material

Survey File

Table 1 Data

The Supplementary materials are available at https://doi.org/10.20935/AcadMed7773.

## Figures and Tables

**Table 1 •:** Demographic of participants.

	IBD *N* = 106 N (%)	Control *N* = 33 N (%)	*p* *
Gender			
Female	58 (54.7)	22 (67)	0.225
Male	48 (45.3)	11 (33.3)	0.225
Age			
18–29	8 (7.6)	1 (3.3)	0.686
30–49	50 (47.6)	12 (4.0)	0.275
>50	47 (44.8)	17 (56.7)	0.470
Education			
Highschool	7 (6.6)	4 (12.2)	0.291
College or higher	99 (93.4)	29 (87.9)	0.291
Race			
White	94 (88.7)	30 (90.9)	1
African American	9 (8.5)	3 (9.1)	1
Other	3 (2.8)	1 (3.0)	0.5488
Pre-Tax Income			
<$20,000	10 (9.4)	2 (6.1)	0.731
$20,001–$100,000	52 (49.1)	22 (66.7)	0.076
>$100,000	30 (28.3)	4 (12.1)	0.066
I prefer not to say	14 (13.2)	4 (15.2)	1
Employment Status			
Employed full time (>39 hours/week)	62 (58.5)	18 (54.5)	0.160
Employed part time (<39 hours/week)	13 (12.3)	4 (12.1)	1
Unemployed	0 (0)	1 (3.0)	0.237
Student	3 (2.8)	0 (0)	1
Retired	15 (14.2)	7 (21.2)	0.331
Homemaker	3 (2.8)	2 (6.1)	0.592
Self-employed	6 (5.7)	0 (0)	0.335
Unable to work	4 (3.8)	1 (3.0)	1

* Fisher’s exact or Chi-square statistics *p*. IBD = Inflammatory bowel disease; CBD = Cannabidiol.

**Table 2 •:** Knowledge, access, and personal usage of cannabis and CBD oil.

	IBD *N* = 106 N (%)	Control *N* = 33 N (%)	*p* *
Have you ever used any of the following?			
Cannabis	57 (53.8)	15 (45.5)	0.404
CBD oil	43 (40.6)	21 (63.6)	0.020 **
Opioids	43 (40.6)	8 (24.2)	0.089
Advil/Tylenol/Ibuprofen	95 (89.6)	31 (93.9)	0.733
Alcohol	76 (71.7)	23 (69.7)	0.825
Do you know what cannabis refers to?			
Yes	105 (99.1)	32 (97)	0.420
Do you know what CBD oil is?			
Yes	101 (95.3)	33 (100)	0.339
Do you have access to cannabis?			
Yes	45 (42.5)	13 (39.4)	0.756
Do you have access to CBD oil?			
Yes	57 (53.8)	19 (53.8)	0.702
Have any of your medical professionals/providers recommended or informed you of the use of cannabis or CBD oil for medicinal purposes?			
Yes	20 (18.9)	3 (9.1)	0.283

* Fisher’s exact or Chi-square statistics *p*.

** Significance at *p* < 0.05. IBD = Inflammatory bowel disease; CBD = Cannabidiol.

**Table 3 •:** Age of initiation and frequency of cannabis or CBD oil use.

	IBD *N* = 62 N (%)	Control *N* = 21 N (%)	*p* *
At what age did you start using cannabis			
<18 years old	16 (25.8)	3 (14.3)	0.374
18–30 years old	28 (45.2)	9 (42.9)	0.572
>30 years old	16 (25.8)	9 (42.9)	0.141
I have never used cannabis or CBD oil	0 (0)	0 (0)	1
How often do you consume cannabis or use CBD oil?			
Less that once per day	22 (35.5)	10 (47.6)	0.323
Once per day	6 (9.7)	3 (14.3)	0.686
Twice per day	4 (6.5)	1 (4.8)	1
Three or more times per day	6 (9.7)	2 (9.5)	1
I do not use cannabis or CBD oil	23 (37.1)	4 (19.0)	0.179

* Fisher’s exact or Chi-square statistics *p*. IBD = Inflammatory bowel disease; CBD = Cannabidiol.

**Table 4 •:** Symptom relief from cannabis and CBD oil use in CD and UC participants.

	CD N (%)	UC N (%)	*p* *
Reported relief from cannabis use: ‘some’, ‘majority’, or ‘complete’ symptom relief	*N* = 41	*N* = 11	
Abdominal pain	27 (65.9)	2 (18.2)	0.007 **
Other pain	32 (78)	5 (45.5)	0.058
Stress ***	32 (80)	5 (45.5)	0.058
Anxiety	29 (70.7)	2 (18.2)	0.004 **
Depression	23 (56.1)	1 (9.1)	0.007 **
Diarrhea	18 (43.9)	2 (18.2)	0.170
Constipation	6 (39)	2 (18.2)	0.671
Headaches	16 (39)	2 (18.2)	0.291
Nausea/vomiting	23 (56.1)	0 (0)	0.001
Allergic reaction	4 (9.8)	0 (0)	0.567
Lack of energy/fatigue	12 (29.3)	2 (18.2)	0.705
Reported relief from CBD oil use: ‘some’, ‘majority’, or ‘complete’ symptom relief	*N* = 39	*N* = 11	
Abdominal pain	16 (41)	0 (0)	0.010 **
Other pain ***	24 (58.5)	5 (45.5)	0.491
Stress	17 (43.6)	2 (18.2)	0.170
Anxiety	15 (38.5)	1 (9.1)	0.080
Depression	13 (33.3)	1 (9.1)	0.148
Diarrhea	11 (28.2)	0 (0)	0.093
Constipation	3 (7.7)	0 (0)	1
Headaches	9 (23.1)	0 (0)	0.177
Nausea/vomiting	11 (28.2)	0 (0)	0.093
Allergic reaction	2 (5.1)	0 (0)	1
Lack of energy/fatigue	9 (23.1)	0 (0)	0.177

Excludes participants who reported indeterminate IBD (*n* = 14).

* Fisher’s exact statistics *p*.

** Significance at *p* < 0.05.

*** Calculated based on 40 and 41 responses, respectively. CD = Crohn’s Disease; UC: ulcerative colitis; CBD = Cannabidiol.

**Table 5 •:** Reported effects on weight and appetite due to cannabis and CBD oil.

	IBD *N* = 62 N (%)	Control *N* = 21 N (%)	*p* *
Which of the following effects has cannabis or cannabidiol (CBD) oil use had for you?			
Caused weight gain	13 (21)	1 (4.8)	0.104
Caused weight loss	1 (1.6)	1 (4.8)	0.444
Increased appetite	39 (62.9)	8 (38.1)	0.047 **
Decreased appetite	0 (0)	0 (0)	1.000

* Fisher’s exact or Chi-square statistics *p*.

** Significance at *p* < 0.05. IBD = Inflammatory bowel disease; CBD = Cannabidiol.

**Table 6 •:** Self-reported effects of cannabis or CBD oil use on opioid dosage and intestinal health among IBD patients.

	IBD *N* = 62 N (%)	*p* *
Which of the following effects has cannabis or CBD oil use had for you?		
Decrease opioid dosage/Induced remission for IBD	21 (33.9)	<0.00001 **
Increase opioid dosage/Caused or increased intestinal side effects	2 (3.2)	<0.00001 **
No effect	46 (74.2)	

* Fisher’s exact or Chi-square statistics compared against “no effect”.

** Significance at *p* < 0.05. IBD = Inflammatory bowel disease; CBD = Cannabidiol.

## Data Availability

Data supporting these findings are available within the article, at https://doi.org/10.20935/AcadMed7773, or upon request.
