# Supplementary material for: Inflammatory bowel disease patients believe cannabis and cannabidiol oil relieve symptoms
Source: Acad Med (San Franc). Author manuscript; Available in PMC 2025 Jul 20. (PMC12276967; doi:10.20935/acadmed7773)
Supplement: Survey File [file NIHMS2091998-supplement-Survey_File.pdf]

Research Study on the Role of Cannabis and Cannabidiol as an Alternative Therapy for Digestive Health

Please proceed with this survey. Thank you!

You are invited to participate in a survey on:
The effect of Cannabis as an alternative treatment and practices in Inflammatory Bowel Disease patients. You are invited to participate in an ANONYMOUS voluntary research survey study. People aged 18 to 69 years with or without a diagnosis of inflammatory bowel disease are eligible to participate in the study. This survey study is being conducted by Abigail Basson, PhD., RD., LD., Dr. Jeffry Katz, MD and Dr. Vu Nguyen, MD from the Digestive Health Research Institute, School of Medicine, Case Western Reserve University.

What is the purpose of this study?

The purpose of this study is to perform a one-time online survey sent via email to determine the knowledge, perception and beliefs of patients with IBD and healthy controls on the role of cannabis and cannabidiol as an alternative therapy for digestive health perception, through a survey sent from REDCap via email.

What do I have to do?

Please answer a ONE-TIME ONLINE QUESTIONNAIRE that is intended to determine the participant's perceptions on the role of cannabis and cannabinoid (CBD) oil on digestive symptoms. The survey contains 4 sections with 37 questions (multiple choice) and it should take approximately 10-15 minutes to complete. No personal information will be collected via the survey.

Do I have to take part?

Your participation in this survey is voluntary. You may refuse to consent in the recruitment (introductory) email or exit the survey at any time without penalty. You are free to decline to answer any question you do not wish to answer for any reason. We anticipate a minimum target enrollment of 100 per group (one group for IBD patients, and another for healthy population)which equates to a total minimum of 200 people total will be enrolled at the UHMC Digestive Health Institute.

What are the possible benefits of taking part?

There will be no direct benefit to the participants in this research study. Your participation in this study might help us understand the knowledge, perception/ beliefs and practices of IBD patients and healthy controls on the role of cannabis and cannabidiol as an alternative therapy for digestive health.

What are the possible risks of taking part?

There is no physical risk to being in this study. There is always the risk that your information may be seen by someone who shouldn't have access. This survey is completely anonymous and no identifying information will be collected at any time. We will store all electronic data on a password-protected, UH-encrypted computer. Since cannabis use may be illegal in your state of residence, there is a very small risk of use becoming known if a breach occurred - we are protecting against this risk by not gathering any identifying information (this is an anonymous survey).

What are the alternatives to taking part?

The alternative to participation in this study is to NOT participate. This will not affect your care in any way.

What will it cost to participate in this study?

Your participation in this study will not involve a cost to you or your insurance company. You will not be paid for participation.

Will taking part in this study be kept confidential?

This survey is completely anonymous and no identifying information will be collected at any time. Your survey answers will be collected within UH RedCap where data will be stored in a password protected electronic format.

Your rights as a participant in a research study

Your participation in this research study is voluntary. Refusing to participate will not alter your usual health care or involve any penalty or loss of benefits to which you are otherwise entitled. If you decide to join the study, you may withdraw at any time and for any reason without penalty or loss of benefits. De-identified data from this study may be published, presented, or otherwise made publicly available. If this happens, your identity will not be revealed.

Students and Employees

Choosing not to participate or withdrawing from this study will not affect your employment or class standing, nor will the results be shared with your supervisors or professors.

Contact Information

If you have questions at any time about the study or the procedures, you may contact Abigail Basson, RD, Ph.D., via phone at 856-220-3445 or via email at Abigail.basson@uhhospitals.org. If you have any questions, concerns, or complaints about the study in the future, you may also contact them later.

If the researchers cannot be reached, or if you would like to talk to someone other than the researcher(s) about; concerns regarding the study; research participant's rights; research-related injury; or other human subject issues, please call the University Hospitals Cleveland Medical Center's Research Subject Rights phone line at (216) 983-4979 or write to: The Associate Chief Scientific Officer, The Center for Clinical Research, University Hospitals Cleveland Medical Center, 11100 Euclid Avenue, Lakeside 1400, Cleveland, Ohio, 44106-7061.

CONSENT TO COMPLETE THE SURVEY

Clicking on the "Yes" option indicates that you agree to the following:

• You have read the above information
• You voluntarily agree to participate
• You are 18 years of age or older

note that completion of the survey implies consent.

◯ Yes
◯ No

SECTION 1: BELIEFS ON CANNABIS PLEASE REMEMBER THAT YOUR RESPONSES WILL BE ANONYMOUS

Do you know what cannabis refers to?

◯ Yes, it is a TV show
◯ Yes, it comes from the marijuana (weed) plant
◯ Yes, it is a type of prescription steroid
◯ No, I do not know what it is

Do you know what cannabidoil (CBD) oil is?

◯ Yes, it is a TV show
◯ Yes, it is a type of steroid
◯ Yes, it comes form the marijuana (weed) plant
◯ No, I do not know what it is

Do you have access to cannabis?

◯ Yes
◯ No
◯ I do not know

Do you have access to cannabidoil (CBD) oil?

◯ Yes
◯ No
◯ I do not know

Have any of your medical professionals/providers recommended or informed you of the use of cannabis or CBD oil for medicinal purposes?

◯ Yes
◯ No
◯ I do not know

Which of the following statements do you believe are true about Cannabis use? select all that apply

☐ It is bad for my health
☐ It is against my religion
☐ It leads to addiction
☐ It is good for my health/mental well-being
☐ None of the above
(select all that apply)

To what extent, on a scale of 1 to 5, do you believe any of the following have a BENEFICIAL effect in relieving symptoms of inflammatory bowel disease?

	1 - Not beneficial	2 - Slightly beneficial	3 - Somewhat beneficial	4 - Very Beneficial	5 - Extremely Beneficial	Not sure
Cannabis (marijuana)	◯	◯	◯	◯	◯	◯
Cannabidiol oil (CBD)	◯	◯	◯	◯	◯	◯
Corticosteroids	◯	◯	◯	◯	◯	◯
Biologics/immunosuppressants	◯	◯	◯	◯	◯	◯

How important do you think medical marijuana is for SYMPTOM RELIEF in patients with inflammatory bowel disease?

◯ 1 - Not important
◯ 2
◯ 3
◯ 4
◯ 5 - Very Important
◯ I do not know

What do you believe cannabis or cannabidiol (CBD) oil could be used for? (select all that apply)

	Medical use	Recreation al	Depression or stress relief	Pain relief	Hair regrowth	Reduce IBD symptoms	None
Cannabis	☐	☐	☐	☐	☐	☐	☐
Cannabidiol (CBD) oil	☐	☐	☐	☐	☐	☐	☐

On a scale of 1 to 5, how strongly would you consider/support using cannabis or cannabidiol (CBD) oil for relieving your IBD symptoms?

◯ 1 - Strongly support
◯ 2
◯ 3
◯ 4
◯ 5 - Do not support
◯ Not sure

On a scale of 1 to 5, how strongly would you consider/support using cannabis or cannabidiol (CBD) oil as a replacement for prescribed opioids?

◯ 1 - Strongly support
◯ 2
◯ 3
◯ 4
◯ 5 - Do not support
◯ Not sure

Have you ever used any of the following? select all that apply

- ☐ Cannabis
- ☐ Cannibidiol (CBD) oil
- ☐ Opioids
- ☐ Advil/Tylenol/Ibuprofin
- ☐ Alcohol
- ☐ I have never used any of the above
- ☐ I prefer not to answer

(select all that apply)

Which of the following purposes have you ever used cannabis or cannibidiol (CBD) oil oil for? (select all that apply)

	Medical use	SHORT term IBD symptom relief	LONG term IBD symptom relief	Pain relief	Anxiety, stress, or depression relief	Recreation al	None of these
Cannabis	☐	☐	☐	☐	☐	☐	☐
Cannabidiol (CBD) oil	☐	☐	☐	☐	☐	☐	☐

Please note any other purposes (not stated above) that you have used cannabis or cannibidiol (CBD) oil for. leave blank if not applicable

____

At what age did you start using cannabis or cannibidiol (CBD) oil?

- ◯ < 18 years old
- ◯ 18-25 years old
- ◯ 26-30 years old
- ◯ > 30 years old
- ◯ I have never used cannabis or CBD oil
- ◯ I prefer not to answer

How often do you consume cannabis or use cannibidiol (CBD) oil?

- ◯ Less than once per day
- ◯ Once per day
- ◯ Twice per day
- ◯ Three or more times per day
- ◯ I do not use cannabis or CBD oil
- ◯ I prefer not to answer

In the past 30 days, how many times have you used/consumed cannabis or cannibidiol (CBD) oil?

____

How do you use or consume cannabis or cannibidiol (CBD) oil? select all that apply

- ☐ Smoking
- ☐ Inhalation
- ☐ Food/edible product
- ☐ Sublingual spray
- ☐ CBD oil/product
- ☐ I do not consume cannabis or CBD oil
- ☐ I prefer not to answer

(select all that apply)

Please note any other forms of cannabis (not stated above) that you have used or consumed. leave blank if not applicable

____

On a scale of 1 to 5, what effect does CANNABIS have for you on relieving any of the following:

	1 - No relief	2 - Minimal relief	3 - Some relief	4 - Majority relief	5 - Complete relief	I do not suffer with this
Abdominal pain	○	○	○	○	○	○
Other pain	○	○	○	○	○	○
Stress	○	○	○	○	○	○
Anxiety	○	○	○	○	○	○
Depression	○	○	○	○	○	○
Diarrhea	○	○	○	○	○	○
Constipation	○	○	○	○	○	○
Headaches	○	○	○	○	○	○
Nausea/vomiting	○	○	○	○	○	○
Allergic reaction	○	○	○	○	○	○
Lack of energy/fatigue	○	○	○	○	○	○

On a scale of 1 to 5, what effect does CANNIBIDIOL (CBD) OIL have for you on relieving any of the following:

	1 - No relief	2 - Minimal relief	3 - Some relief	4 - Majority relief	5 - Complete relief	I do not suffer with this
Abdominal pain	○	○	○	○	○	○
Other pain	○	○	○	○	○	○
Stress	○	○	○	○	○	○
Anxiety	○	○	○	○	○	○
Depression	○	○	○	○	○	○
Diarrhea	○	○	○	○	○	○
Constipation	○	○	○	○	○	○
Headaches	○	○	○	○	○	○
Nausea/vomiting	○	○	○	○	○	○
Allergic reaction	○	○	○	○	○	○
Lack of energy/fatigue	○	○	○	○	○	○

Which of the following effects has cannabis or cannabidiol (CBD) oil use had for you? select all that apply

- ☐ Caused weight GAIN
- ☐ Caused weight LOSS
- ☐ INCREASED appetite
- ☐ DECREASED appetite
- ☐ None of the above
- ☐ I do not use cannabis or CBD oil

(select all that apply)

Which of the following effects has cannabis or cannabidiol (CBD) oil use had for you? select all that apply

- ☐ DECREASE my opioid dosage (e.g. oxycodone)
- ☐ INCREASE my opioid dosage (e.g. oxycodone)
- ☐ Induced remission for my IBD
- ☐ Caused or INCREASED intestinal side effects
- ☐ None of the above
- ☐ I do not use cannabis or CBD oil

(select all that apply)

Please note any other effects (not stated above) you have noticed with cannabis or cannibidiol (CBD) oil use. leave blank if not applicable	__________________________

SECTION 2: ABOUT YOURSELF

Do you suffer from ANY of the following? select all that apply	☐ Crohn's disease<br>☐ Ulcerative colitis<br>☐ Indeterminate inflammatory bowel disease<br>☐ Irritable bowel syndrome (IBS)<br>☐ Diabetes<br>☐ Cancer<br>☐ None of the above<br>(select all that apply)
How long ago were you diagnosed with Crohn's disease or ulcerative colitis?	○ < 1 year ago<br>○ 1-5 years ago<br>○ 6-10 years ago<br>○ > 11 years ago<br>○ I have never been diagnosed with these
Have you suffered from any of the following in the last TWO WEEKS? select all that apply	☐ Abdominal pain/cramping<br>☐ Diarrhea<br>☐ Constipation<br>☐ Fatigue<br>☐ Weight loss<br>☐ Physical pain lasting more than 48 hours<br>☐ I have not suffered from any of these<br>(select all that apply)
Have you suffered from any of the following in the last SIX MONTHS? select all that apply	☐ Abdominal pain/cramping<br>☐ Diarrhea<br>☐ Constipation<br>☐ Fatigue<br>☐ Weight loss<br>☐ Physical pain lasting more than 48 hours<br>☐ I have not suffered from any of these<br>(select all that apply)
On a scale of 1 to 5, how would you rate your mental health in the past? select all that apply	○ Poor<br>○ Fair<br>○ Good<br>○ Very Good<br>○ Excellent<br>○ I prefer not to answer

What US state do you live in?

- ◯ Alabama
- ◯ Alaska
- ◯ Arizona
- ◯ Arkansas
- ◯ California
- ◯ Colorado
- ◯ Connecticut
- ◯ Delaware
- ◯ Florida
- ◯ Georgia
- ◯ Hawaii
- ◯ Idaho
- ◯ Illinois
- ◯ Indiana
- ◯ Iowa
- ◯ Kansas
- ◯ Kentucky
- ◯ Louisiana
- ◯ Maine
- ◯ Maryland
- ◯ Massachusetts
- ◯ Michigan
- ◯ Minnesota
- ◯ Mississippi
- ◯ Missouri
- ◯ Montana
- ◯ Nebraska
- ◯ Nevada
- ◯ New Hampshire
- ◯ New Jersey
- ◯ New Mexico
- ◯ New York
- ◯ North Carolina
- ◯ North Dakota
- ◯ Ohio
- ◯ Oklahoma
- ◯ Oregon
- ◯ Pennsylvania
- ◯ Rhode Island
- ◯ South Carolina
- ◯ South Dakota
- ◯ Tennessee
- ◯ Texas
- ◯ Utah
- ◯ Vermont
- ◯ Virginia
- ◯ Washington
- ◯ West Virginia
- ◯ Wisconsin
- ◯ Wyoming

What effect did your state's legalization of cannabis have on your use of cannabis or cannibidiol (CBD) oil?

- ◯ DECREASED my use of cannabis or CBD oil
- ◯ INCREASED my use of cannabis or CBD oil
- ◯ No effect (my use stayed the same)
- ◯ I prefer not to answer
- ◯ I have never used cannabis or CBD oil

What is your AGE?	◯ 18-21<br>◯ 22-25<br>◯ 26-29<br>◯ 30-35<br>◯ 36-39<br>◯ 40-49<br>◯ 50-59<br>◯ 60-69<br>◯ Prefer not to say
To which GENDER do you most identify?	◯ Female<br>◯ Male<br>◯ Transgender female<br>◯ Transgender male<br>◯ Gender Variant/Non-conforming<br>◯ Non-binary<br>◯ None of the above<br>◯ Prefer not to say
How would you describe yourself? select all that apply	☐ American Indian or Alaska Native<br>☐ Asian<br>☐ Black or African American<br>☐ Hispanic<br>☐ Native Hawaiian or other Pacific Islander<br>☐ White Non-Hispanic<br>(select all that apply)
Are you of Hispanic, Latino, or Spanish origin?	◯ Yes<br>◯ No<br>◯ Prefer not to say
What is the highest degree or level of school you have completed?	◯
What is your current employment status?	◯ Employed full time (> 39 hours/week)<br>◯ Employed part time (< 39 hours/week)<br>◯ Unemployed and looking for work<br>◯ Unemployed and not looking for work<br>◯ Student<br>◯ Retired<br>◯ Homemaker<br>◯ Self-employed<br>◯ Unable to work
What is your current pre-tax income?	◯ < $20,000<br>◯ $20,001-$40,000<br>◯ $40,001-$60,000<br>◯ $60,001-$80,000<br>◯ $80,001-$100,000<br>◯ >$100,000<br>◯ I prefer not to say

If you are struggling with a substance use disorder or addiction, there are many resources available to help.

Call SAMHSA's National Helpline at 1-800-662-HELP (4357) or TTY: 1-800-487-4889, or text your zip code to 435748 (HELP4U), or use FindTreatment.gov to get help.

The USDEA also has various recovery resources as listed in the following webpage: https://www.dea.gov/recovery-resources.

Thank you for taking our survey!
Please click "Submit" and close this browser to exit the survey.
